# Accurate Physical Property Predictions via Deep Learning

**DOI:** 10.3390/molecules27051668

**Published:** 2022-03-03

**Authors:** Yuanyuan Hou, Shiyu Wang, Bing Bai, H. C. Stephen Chan, Shuguang Yuan

**Affiliations:** 1 Research Center for Computer-Aided Drug Discovery, Shenzhen Institute of Advanced Technology, Chinese Academy of Sciences, Shenzhen 518055, China; yy.hou@siat.ac.cn (Y.H.); sy.wang@siat.ac.cn (S.W.); bing.bai@siat.ac.cn (B.B.); 2 Biomedicial Department, University of Chinese Academy of Sciences, Beijing 100049, China; 3 AlphaMol Science Ltd., Shenzhen 518055, China

**Keywords:** aqueous solubility, oil–water partition coefficient, logS, logP, logD, deep learning, SMILES enumeration

## Abstract

Neural networks and deep learning have been successfully applied to tackle problems in drug discovery with increasing accuracy over time. There are still many challenges and opportunities to improve molecular property predictions with satisfactory accuracy even further. Here, we proposed a deep-learning architecture model, namely Bidirectional long short-term memory with Channel and Spatial Attention network (BCSA), of which the training process is fully data-driven and end to end. It is based on data augmentation and SMILES tokenization technology without relying on auxiliary knowledge, such as complex spatial structure. In addition, our model takes the advantages of the long- and short-term memory network (LSTM) in sequence processing. The embedded channel and spatial attention modules in turn specifically identify the prime factors in the SMILES sequence for predicting properties. The model was further improved by Bayesian optimization. In this work, we demonstrate that the trained BSCA model is capable of predicting aqueous solubility. Furthermore, our proposed method shows noticeable superiorities and competitiveness in predicting oil–water partition coefficient, when compared with state-of-the-art graphs models, including graph convoluted network (GCN), message-passing neural network (MPNN), and AttentiveFP.

## 1. Introduction

Accurate prediction of molecular properties would offer reliable guidance in profiling lead compounds in the drug-discovery process. The traditional drug design workflows, often biased by the experiences of chemists, rely on time-consuming and expensive simulations and experiments to acquire the relevant molecular properties [1]. Launching a novel drug into the market would take more than ten years on average, with a substantial investment of billions of USD [2]. Meanwhile, deep learning shows great success in other fields, such as natural-language processing [3,4,5,6,7,8,9] and pattern recognition [10,11,12,13], as well as the improvement of computing power and dataset availability. Its potential in promoting efficiency and success rate of drug development, in particular the prediction of molecular properties, has been widely investigated for years [14,15,16,17,18]. The current mainstream algorithms for molecular characterization can be simply divided into two categories—a graph model based on molecular graphs, or a sequence model based on SMILES (Simplified Molecular-Input Line-Entry System) [19] sequence input. A molecular graph is a two-dimensional representation of a chemical molecule, and accounts for its topo-structural features and atom connectivity (e.g., adjacency). Graph-based learning methods have been widely developed in the field of drug development [17,20,21,22,23,24,25]. Various graph neural network (GNN) [26] variants have also demonstrated their effectiveness in capturing inter-node relationships through message-passing between graph nodes. For example, Gilmer et al., summarized different variants and applications of a message-passing neural network (MPNN) in quantum chemistry calculations [20]. Schütt et al. [25] proposed a Continuous-Filter Convolutional Neural Network modeling quantum interactions in molecules, and AttentiveFP [21] proposed a new type of GNN with graph attention mechanism suitable for molecular characterization. The latter has the best prediction expression on a drug-discovery-related dataset. GNNs are therefore considered to be an attractive modeling method for molecular property prediction.

The SMILES characterization is very popular among chemists and machine-learning researchers. It has been widely used in learning-based analyses in drug discovery [14,27,28,29]. Segler et al. [27] demonstrated that recurrent neural network (RNN) trained on molecular SMILES strings would be able to learn the grammar of language as well as the distribution of chemical space. Analogous to a text stream, each symbol in SMILES can be regarded as a word. Inspired by the word2vec [3] technology in natural-language processing, the smiles2vec [14] technology similarly processes the SMILES sequences, in which specific combinations of SMILES elements are transformed into alternative representations via a pre-training method. The new representations are then mapped against a pre-defined dictionary for downstream tasks. A neural network combined with smiles2vec shows superior performance in predicting distinct chemical properties, including toxicity, activity, solubility and solvation energy. Meanwhile, transformer [6] becomes another popular class of neural network in natural-language processing. Notable examples include seq2seq [9] and the Bidirectional Encoder Representations from Transformers (BERT) [7]. Due to the similarity between chemical language and natural language, researchers in the field of molecular characterization have also introduced transformer-based large models plus pre-trained and fine-tuned models, and obtained good results in chemical reaction prediction [30] and other fields.

In this study, we exploit the three advantages of SMILES strings over molecular graphs. First, linear strings are generally more compact than graph formats, and comprise only the crucial information for defining the chemistry of a molecule. Second, a single molecule may have multiple possible SMILES strings. For example, CCO, OCC and C(O)C all specify ethanol. The enumeration of characters in SMILES string can be achieved more easily, when compared to the generation of isomorphous graphs. This characteristic is particularly useful for data augmentation, when a dataset generally contains few samples. Finally, SMILES strings carry characters that explicitly indicate substructures and other topological information, such as branching, looping structures, and chirality [31]. Here, we selected SMILES strings as molecular inputs for deep learning, and constructed a Bidirectional long short-term memory with Channel and Spatial Attention network (BCSA). BCSA is based on Bidirectional Long Short-Term Memory (BILSTM) [32], followed by channel attention and spatial attention modules. Data augmentation of molecular structures was achieved by SMILES enumeration [33], to acquire more tokens as inputs for our models. Generalization error was reduced substantially by taking the average prediction of all enumerated SMILES for a given molecule. Due to its importance among the physiochemical properties of a drug molecule, aqueous solubility prediction has been a subject of intensive studies for many years [15,34,35,36,37,38,39,40,41,42]. Hence, we applied our model and workflow first in predicting water solubility and later extended into the prediction of oil–water partition coefficient. The prediction performance was compared against three other state-of-the-art graphs models, to evaluate its accuracy, generalizability and reliability.

## 2. Results

### 2.1. Training of BCSA Model

**Figure 1:**
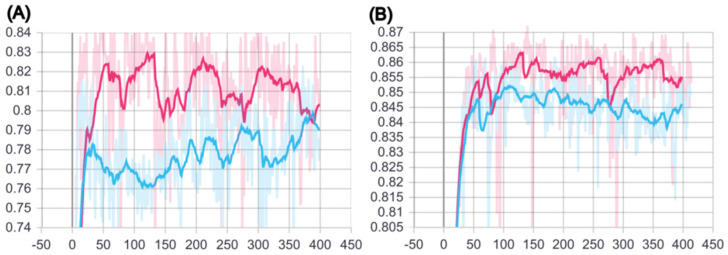
$R^{2}$ curves of the BILSTM model (blue line) and BCSA model (red line) in (**A**) the validation set, and (**B**) of the test set.

We aimed to develop a new deep-learning architecture using SMILES sequence auto-encoder and explored the role of predicting molecular solubility and other properties. We trained different datasets including training set (7955), validation set (996) and testing set (995). Then, we performed training on the best hyperparameter (Table 1 using both BILSTM model and our BCSA model. Figure 1A indicated the trend of $R^{2}\;$when we trained on validation sets with 400 epochs. Figure 1B showed the trend of $R^{2}$ when we trained on testing sets with 400 epochs. Moreover, the smoothing parameters of the curve is set to approximately 0.8, to reduce the noise. Figure 1 clearly shows that our model has stronger fitting effect and generalizability than the BILSTM model on both the validation and testing set.

A general way to improve a deep-learning model is to increase the size of a dataset. Dataset augmentation offers more training data and becomes a particularly effective technique in the field of images. The diversity of SMILES string would generate more new data points. All SMILES strings used in this work were augmented using the SMILES enumeration (SE) technique. Two models were trained with the dataset size up to 20× and 40× of the original segmented dataset (each molecule has 20 and 40 different SMILES representations respectively). Since simple molecules may have less than 20 distinct SMILES string variants, identical variants may be generated in the augmentation step and these duplicates were removed to avoid potential duplicate bias. The numbers of data points between training, validation and testing for the 20× and 40× datasets were 134,454:19,881:16,834 and 239,260:30,042:39,800, respectively. The model with the best performance of $R^{2}$ in the training process was taken forward to the validation sets. Table 2 shows the performance results of test datasets by taking the average predicted value for each molecule obtained from the enumerated SMILES. We found that both the stability and generalization ability of the enumerated model were significantly improved. The best result was achieved in the SMILES × 40 datasets, indicating that the enumerated model better paid attention to the different sequence information of the molecules. In contrast, we achieve significant performance improvement for the Cui datasets which had $R^{2}$ = 0.72–0.79 with RMSE = 0.988–1.151 [15], whereas the test performance of in this work is $R^{2}$ = 0.83–0.88 with RMSE = 0.79–0.95. This model will be further trained to with more dataset to improve the final accuracy.

### 2.2. Compare with State-of-the-Art Models

**Figure 2:**
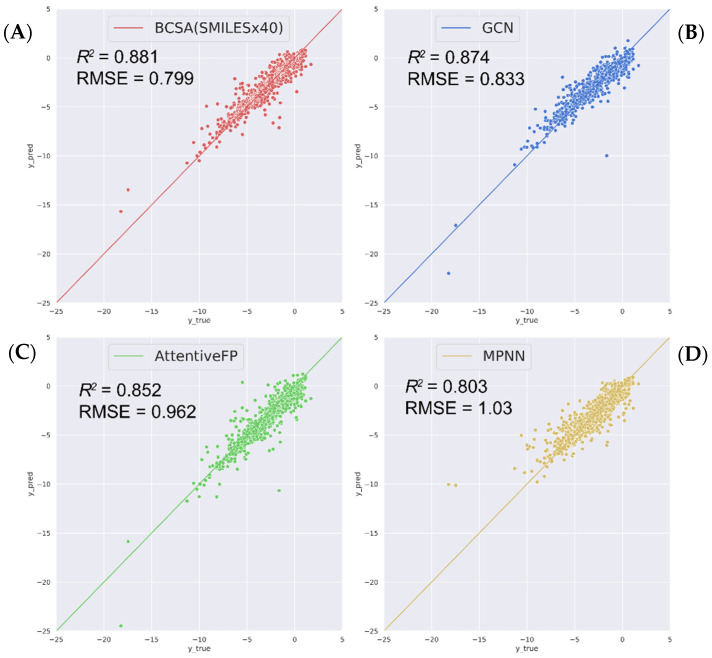
Scatter plots of the predicted log solubilities of four different model. (**A**) BCSA model with SMILES enumeration, (**B**) GCN model on source canonical SMILES, (**C**) AttentiveFP model on source canonical SMILES, and (**D**) MPNN model on source canonical SMILES. The diagonal line in each plot denotes a perfect correlation (*y* = *x*).

To better demonstrate the competitiveness of our sequence-based model, its performance in solubility prediction was compared with other state-of-the-art graph models, including GCN [43], MPNN [20], AttentiveFP [21]. The framework was implemented using python software package dgl-Lifesci [44] (the training hyperparameters detailed in Supporting Information: Tables S1–S3). Figure 2 shows the predicted value against the experimental value from the original test datasets. The predicted values of a better model would populate more closely to the diagonal line (*y* = *x*). All four tested models demonstrate excellent predictive abilities. Among them, our BCSA model with 40× data augmentation achieves the best performance on molecular solubility prediction, reflected by a better correlation and a smaller deviation from the experimental data.

### 2.3. Predicting Other Related Physicochemical Properties

**Figure 3:**
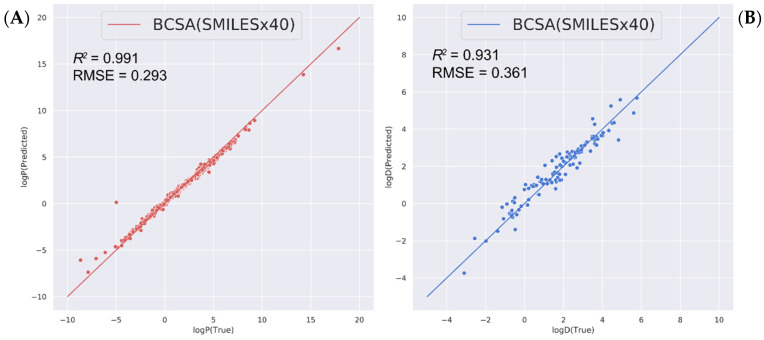
Scatter plots for (**A**) logP and (**B**) logD predictions with our BCSA model (SMILES × 40).

We extended the predictions of other relevant molecular properties, namely the oil–water partition coefficients logP and logD (pH = 7.4), with our BCSA (SMILES × 40) model (the training hyperparameters detailed in Supporting Information: Tables S4 and S5). The logP dataset is still based on the Cui et al. [15] dataset. As shown in Figure 3A, our model achieves an exciting result in the test dataset, with $R^{2}$ of 0.99 and *RMSE* of 0.29. The scatter plot demonstrates that the predicted data achieves excellent fitting throughout the whole range of the experimental logP values. Meanwhile, the logD (pH = 7.4) training dataset was taken from Wang et al. [45]. The dataset is randomly divided into 8:1:1. The training data are obtained using SMILES enumeration [33] 40×. Eventually, the 40× dataset in a 31,290:3858:4031(TRAIN: VAL: TEST) ratio was obtained. The average predicted logD values of each molecule was chosen as the final prediction result. Our model shows a $R^{2}$ = 0.93 with RMSE = 0.36 in the testing set (Figure 3B), whereas support vector machine (SVM) models by Wang et al., shows $R^{2}$ = 0.89 with RMSE = 0.56 for the testing set. Apparently, our model outperforms that by Wang et al. Moreover, our model also shows better performance for oil–water-related predictions. The results indicated that our model could give a reliable and robust prediction.

In summary, introducing the two attention modules significantly improves the prediction accuracy $R^{2}\;$by 5% in both the verification set and the test set, when compared with pure BILSTM. Moreover, the BILSTM with attention model resulted in larger variances between the predicted and the ground-truth values, a sign of possible overfitting often caused by a small dataset. Therefore, the dataset in this study was enriched by enumeration of SMILES for each molecule. Our result clearly shows that the accuracy, generalizability and overfitting problem are improved with an increasing number of enumerated SMILES strings. Furthermore, our model outperforms three classical graph neural network models (GCN, MPNN, AttentiveFP) in the prediction of aqueous solubility. When trained to predict other relevant properties, logP and logD, our model also appears reliable and reaches prediction accuracy of 0.99 and 0.93, respectively.

## 3. Discussion

For accurate prediction of aqueous solubility, we proposed an end-to-end deep-learning framework, in short BCSA, which combines a BILSTM neural network and the channel and spatial attention modules. By exploiting the advantages of molecular SMILES strings as training inputs, our BCSA model would be able to capture directly the complex spatial information of connected atoms, which has posed a great challenge in previous attempts at the prediction. The overfitting problem arising from small dataset size is also circumvented by SMILES enumeration, which effectively enriches the sample size for training. Successful data augmentation in our workflow would possibly be the reason for its superior accuracy over three other commonly used graph-based neural networks. These networks may be further improved via a similar data enrichment process, during which sufficient isomorphous graphs of each molecule need to be generated for training. It should also be noted that variations on the training dataset may have a strong impact on prediction accuracy. Meanwhile, the channel and spatial attention modules facilitate the identification of influential attributes between adjacent atoms in the SMILES, without incurring greater overhead in computation. Encouragingly, our BCSA model does not require additional auxiliary data for predicting logP and logD with even higher accuracy. The prediction accuracy in terms of $R^{2}\;$is logP > logD > logS. Since most SMILES from the chemical dataset do not offer explicit information of hydrogen (or specifically the ionization and/or tautomerization states), the training input may possess sufficient information for predicting logP, which only accounts for the neutral form of a molecule in both oil and aqueous media. However, the ionized and neutral forms of molecules are effectively different chemical species that cannot be represented with one single SMILES and may have fairly different solubilities. Hence, the prediction performance for logD and logS may further be improved when possible tautomers are considered during the SMILES enumeration step. Nevertheless, with this advanced new algorithm, other properties such as ADMET or DMPK might be potentially predicted accurately as well if the SMILES datasets of the molecule were given. More precisely, our BCSA model, possibly in combination with alternative attention modules, also needs to be evaluated on other datasets in the future for robustness.

## 4. Materials and Methods

### 4.1. Molecular Dataset and Processing

The dataset derived from the work of Cui et al., 2020 [15] contains the 9943 nonredundant compounds. To predict the molecular property value of a compound, its chemical information needs to be represented in a format compatible for machine learning. SMILES format is a common choice for incorporating topological information based on chemical bonding rules. For example, cyclohexane and dioxane may be written as C1CCCCC1 and O1CCOCC1, respectively. As a “chemical language” [19] that encodes the structural information of a molecule into a compact text string under fixed rules and conventions, the SMILES of a molecule comprises simple character(s) for atoms and bonds, among which adjacent characters have high correlation in a chemical sense. Inspired by ref. [30], we tokenized the SMILES strings of drug molecules using the following regular expression:

token_regex = “(\[[^\]]+]Br?|Cl?|N|O|S|P|F|I|b|c|n|o|s|p|\(|\)|\.|=|


#|−|\+|\\\\\/|:|∼|@|\?|>|\*|\$|\%[0–9]{2}|[0–9])”.(1)

By means of word2vec [46,47], an input SMILES sequence is split into n tokens, which are then embedded into a vector of l dimensions according to the token position. Hence, the tokenized characters are eventually embedded into a 2D feature matrix $M\in R^{n\times l}$ before training. Word2Vec would encode tokens into dense vectors by learning the association of the context of the SMILES string. Moreover, the dataset was expanded to SMILES enumeration [33] and the SMILES strings which were padded with “padding” to a fixed length of 150 tokens. The excess characters beyond this length were discarded directly. Finally, the dataset was randomly split into a training (80%) set and validation (10%) set and test (10%) set, respectively.

### 4.2. Model Building

**Figure 4:**
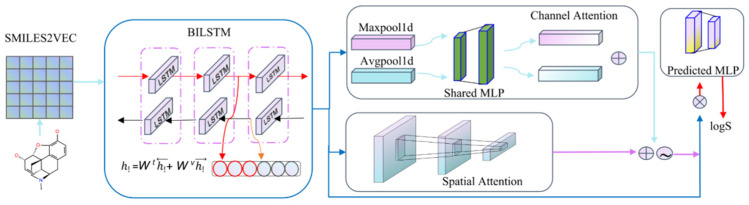
The architecture of the BCSA model. We compress the smiles into vectors via data preprocessing to feed into our trained model. The model consists of three main components: a BILSTM, an improved Convolution Block Attention Module (CBAM) and a predicted MLP network with two fully connected dense layers. The CBAM model contains two parts: channel attention and spatial attention. Both attentions are used in parallel, then add to the outputs which are normalized with sigmoid function to obtain an information-enriched attention map.

Here we provide an overview of the proposed BCSA framework (Figure 4) and introduce the core methods in our model. In addition, we specify the implementation details and the evaluation criteria.

Our model architecture consists of three neural networks. The first one used in this work was based on the Long Short-Term Memory (LSTM) architecture [48] which managed the remote relationship in natural-language processing. Each molecular matrix M is composed of n token vectors which are independent of each other. To obtain the correlation between adjacent tokens, a BILSTM is introduced to process a molecule with two hidden LSTM layers (forwards and backwards), which not only can encode information from front to back, but also obtain information from back to front. The SMILES string can be represented in a sequence of token embeddings as $M=\left[x_{1},x_{2},x_{3},\ldots ,x_{n}\right]$. LSTM creates a hidden state $h_{t}$ by forgetting the hidden state $h_{t-1}$ and remembering new information from molecular embedding $x_{t}$. Adding $\vec{h_{t}}$ and $\overset{\leftarrow }{h_{t}}$ becomes a more informative vector to acquire relationships between adjacent token embeddings in a SMILES string. The output of hidden states in every step t can be defined as:

(2) $$\begin{array}{c} \;\;h_{t}=W^{e}\overset{\leftarrow }{\left(h_{t}\right)}+W^{v}\vec{\left(h_{t}\right)}\\ \;=\vec{\mathrm{LSTM}}\left(x_{t},\vec{h_{t-1}}\right)+\overset{\leftarrow }{\mathrm{LSTM}}\left(x_{t}\overset{\leftarrow }{,h_{t+1}}\right) \end{array}$$

where $W^{e}$ or $W^{v}\;$is the learned weights, all $h_{t}$ can be turned into a simple concatenation $C=\left\{h_{1},h_{2},\ldots ,h_{n}\right\}$. Generally, *C* is a simple representation of the last hidden state of the BILSTM encoder.

The next goal is to locate key tokens or features corresponding to certain parts of the molecule that contributes the most to property prediction. Concretely, this helps uncovering the connections between tokens and predicted value. Chemists can then apply such knowledge to design or improve drug compounds. Therefore, we introduced a convolutional block module attention mechanism in BILSTM. In the second network, we embedded the optimized Convolution Block Attention Module (CBAM) mechanism [49] into the current forward sequential neural network. It has two modules including a channel attention map ($M_{c}$) and a spatial attention map ($M_{s}$), which exploit the inter-channels and inter-spatial relationship of features. In other words, from a spatial viewpoint, the channel attention explores the globality of the molecular hidden state C,while the spatial attention focus on local. Both attentions are used in parallel, then add to the outputs which are normalized with sigmoid function to obtain an information-enriched attention map. The overall attention process can be expressed as:

(3) $$C^{\prime }=\sigma \left(M_{c}\left(C\right)+M_{s}\left(C\right)\right)⊗C$$

where ⊗ denotes element-wise multiplication, σ denotes the sigmoid function, and $C^{\prime }\;$is the output of the overall attention model, respectively. The following introduces the details of each attention module.

Channel attention module: This module focuses on ‘what’ is meaningful given as SMILES tokens. To extract channel attention more efficiently, the dimension of the input hidden layers feature vector C requires further reduction. Woo et al. [49] proposed using both average-pooled and max-pooled features, which greatly improves performance of the model. The training vectors of BILSTM were first aggregated using average-pooling and max-pooling. Two different descriptions were then generated: $C_{avg}$ and $C_{max}$, which represent the average-pooled and max-pooled the training features vectors, respectively. Next, these descriptions were taken forward to a two-layers shared MLP and merging the output of average-pooled vectors ($M_{avg}\left(C\right)$) and max-pooled vectors ($M_{max}\left(C\right)$), using element-wise summation.

(4) $$\;\;M_{c}\left(C\right)=MLP\left(AvgPool1d\left(C\right)\right)+MLP\left(MaxPool1d\left(C\right)\right)\;=W_{1}\left(\sigma \left(W_{0}\left(C_{avg}\right)\right)+W_{1}\left(\sigma (W_{0}\left(C_{max}\right)\right)\right)\;=M_{avg}\left(C\right)+M_{max}\left(C\right)$$

where σ denotes the ReLU function [50] which reduces the overhead of the network, and $W_{0},W_{1}$ are the related MLP weights.

Spatial attention module: This module focuses on the informative part of the SMILES word vector. Here it comprised a two-layers and 1-dimensional Convolutional Neural Networks. The Spatial attention component is computed as

(5) $$\;M_{s}\left(C\right)=Conv1d^{7,1}\left(\sigma \left(Conv1d^{7,16}\left(C\right)\right)\right)$$

where σ denotes the ReLU function, and $Conv1d^{7,x}$ is a 1-D convolution layer with x filters and seven kernels in total. Finally, the attention values were broadcasted accordingly during element-wise multiplication operation, using the following formula:

(6) $$\;O=AvgPool1d\left(\sigma \left(M_{c}\left(C\right)+M_{s}\left(C\right)\right)⊗C\right)\;=AvgPool1d(C^{\prime })$$

where O denotes the aggregated output of the overall attention model by a 1-D average-pooling operation.

In the third network, the abovementioned output vectors O were fed into two fully connected dense layers to predict the molecular property values. ReLU was used as the activation function, which has been widely adopted in deep-learning research. In addition, the dropout layers were also added to avoid the overfitting. The model was trained by minimizing the mean square error (MSE) in a loss function defined as:

(7) $$MSE=\frac{1}{N}\overset{N}{\underset{i=1}{\sum }}{\left({\hat{y}}_{i}-y_{i}\right)}^{2}$$

where ${\hat{y}}_{i}\;$is the predicted value, and $y_{i}$ is the ground-truth solubility values of *N* molecules.

### 4.3. Hyperparameter Search

Several components, known as hyperparameters, were used to control the behavior of the learning algorithm in our model. The performance of the model can fluctuate significantly with different parameters. Here we can use Bayesian optimization [51] to select efficiently the best parameters. During optimization, the target function for minimization was defined as

(8) $$-R^{2}=-(1-(\overset{N}{\underset{i=1}{\sum }}{\left({\hat{y}}_{i}-y_{i}\right)}^{2}/\overset{N}{\underset{i=1}{\sum }}{\left(y_{i}-\bar{y}\right)}^{2})$$

where ${\hat{y}}_{i}$ is the predicted value, $y_{i}$ is the ground-truth value and $\bar{y}$ is the mean value of N molecules. We performed a TPE (Tree-structured Parzen Estimator Approach) search [52] on hyperparameter space as shown in Table 1. In short, 100 models were trained for 60 epochs and an early stop strategy (patience = 20) was set, in order to accelerate training speed. The best hyperparameter space was determined when the model yields $R^{2}\;$value closest to 1 using in the training set (Table 1). The hyperparameters were later used in the validation step. If needed, the accuracy of this model could further be improved by selecting the best hyperparameter set from 30 additionally trained epochs.

The framework was performed using Pytorch and all trainings were finished in a Linux server (openSUSE 15.2): Intel(R) Xeon(R) Platinum 8173M CPU @ 2.00 GHz and Nvidia GeForce RTX 2080 Ti graphics card with 11G. This machine is located internally in the Shenzhen Institute of Advanced Technology, CAS (Shenzhen, China).

### 4.4. Evaluation Metrics

Four performance indicators commonly used in the regression task were chosen to assess our model, including: $R-Squared(R^{2})$, spearman, RMSE, MAE. $R^{2},\;spearman$ can monitor the good-fit capacity of our model to the dataset. Better fitting is expected when these indicators approach 1. RMSE,MAE keep track of the deviations between predicted values and experimental values. Good concordance is achieved where these indicators approach 0.

## Figures and Tables

**Table 1:** Hyperparameters space and the best for model.

Parameter	Possible Values	The Best Found
batch_size	(512,1024)	1024
vocab_size	(120,150)	120
Smiles_max_len	(150,200)	150
hidden_size	(16,32,64)	64
number_layers	3–5	3
dropout	0–0.6	0.12215
mlp_hidden_size	(32,64)	32
learning_rate	0.01–0.001	0.00966

**Table 2:** Statistics of predicted values, values are for validation/Testing set, respectively.

Dataset		(Higher is Better)		(Lower is Better)	
		*R* ^2^	Spearman	*RMSE*	*MAE*
Source data	validation	0.8714	0.9294	0.8085	0.5671
	Test	0.8365	0.9185	0.9513	0.6435
SMILES × 20	validation	0.8790	0.9352	0.8233	0.5512
	Test	0.8779	0.9339	0.8181	0.5493
SMILES × 40	validation	0.8828	0.9375	0.8025	0.5207
	Test	0.8813	0.9361	0.7997	0.5226

## Data Availability

The SMILES enumeration datasets and predicted values are available at https://github.com/summer-cola/SMILES-enumeration-datasets (accessed on 14 January 2022). The logS, logP, logD source datasets are available from the literatures [15,45] and were used as provided.

## Supplementary Materials

The following supporting information can be downloaded online: Table S1: The training hyperparameters of the GCN model; Table S2: The training hyperparameters of the AttentiveFP model; Table S3: The training hyperparameters of the MPNN model; Table S4: The training hyperparameters of logP with the BCSA model; Table S5: The training hyperparameters of logD with BCSA model.

## Sample Availability

Our BCSA models are accessible at http://cadd.siat.ac.cn/molpre/ (accessed on 14 January 2022).
