# Supplementary material for: Accurate Physical Property Predictions via Deep Learning
Source: Molecules. 2022 Mar 3;27(5):1668. doi: 10.3390/molecules27051668 (PMC8912091; doi:10.3390/molecules27051668)
Supplement: Supplementary file 1 [file molecules-27-01668-s001.zip › molecules-1578034-supplementary.pdf]

Supporting Information: Accurate physical property predictions via deep learning

Yuanyuan Hou1,2, Shiyu Wang1, H.C. Stephen Chan1*, Shuguang Yuan1*

[1] Research Center for Computer-aided Drug Discovery, Shenzhen Institute of Chinese Academy of Sciences, Shenzhen, China
[2] University of Chinese Academy of Sciences, Beijing, China
* Correspondence: shuguang.yuan@siat.ac.cn, xc.chen@siat.ac.cn

Supplementary Table S1. The training hyperparameters of the GCN model.

Hyperparameter	Value
learning_rate	0.002
weight_decay	0
patience	30
batch_size	128
dropout	0.05
gnn_hidden_feats	256
predictor_hidden_feats	128
num_gnn_layers	3
residual	True
batchnorm	false
n_tasks	1
atom_featurizer_type	canonical
bond_featurizer_type	canonical
in_node_feats	74

Supplementary Table S2. The training hyperparameters of the AttentiveFP model.

Hyperparameter	Value
learning_rate	0.0003
weight_decay	0
patience	30
batch_size	128
dropout	0
num_layers	3
num_timesteps	2
graph_feat_size	200
n_tasks	1
atom_featurizer_type	canonical
bond_featurizer_type	canonical
in_node_feats	74
In_edge_feats	13

Supplementary Table S3. The training hyperparameters of the MPNN model.

Hyperparameter	Value
learning_rate	0.0003
weight_decay	0
patience	30
batch_size	128
node_out_feats	64
edge_hidden_feats	128
num_step_message_passing	6
num_step_set2set	6
num_layer_set2set	3
n_tasks	1
atom_featurizer_type	canonical
bond_featurizer_type	canonical
in_node_feats	74
In_edge_feats	13

Supplementary Table S4. The training hyperparameters of logP with the BCSA model.

Hyperparameter	Value
batch_size	1024
vocab_size	120
smiles_max_len	200
hidden_size	64
number_layers	3
dropout	0.12215
mlp_hidden_size	32
learning_rate	0.00966

Supplementary Table S5. The training hyperparameters of logD with BCSA model.

Hyperparameter	Value
batch_size	512
vocab_size	120
smiles_max_len	200
hidden_size	64
number_layers	5
dropout	0.41296
mlp_hidden_size	32
learning_rate	0.00708
